# Imperial Microbiology: The National Collection of Type Cultures and the Management of Microorganisms, 1916–1922

**DOI:** 10.1007/s10739-025-09809-8

**Published:** 2025-04-25

**Authors:** James F. Stark

**Affiliations:** University of Leeds, Leeds, UK

**Keywords:** Microorganisms, Collections, Nomenclature, Bacteriology, Ralph St. John-Brooks

## Abstract

Since its founding in January 1920, the National Collection of Type Cultures (NCTC) has played a fundamental role supporting microbiological research in Britain and globally. NCTC is an international repository for authenticated bacterial strains of medical and veterinary significance, making many available to researchers. Among the oldest collections of its kind still operating today, it presently holds almost 6,000 historically and microbiologically significant strains. Drawing on records of the Medical Research Council, which sponsored the NCTC, and uncataloged, previously unstudied archival holdings at the UK Health Security Agency, this article lays out and for the first time critically examines details of the establishment of the NCTC, and explores its far-reaching impacts on microbiology in the 20th century, and particularly on microbial taxonomy and classification.

## Introduction

On December 14,  1934 a dramatic article was published in the provincial British newspaper, the *Hull Daily Mail*. “Our Ceaseless War Against Disease” claimed that scientific research was making unprecedented strides in preventing disease and “driving it back to its last lines of defence” (Anon. 1934). In addition to the “eagle eye” of the Ministry of Health keeping watch over the nation’s health, the article also highlighted the work of four research institutions whose contributions were vital “in the same great work—conquering disease” (Anon. 1934). These were the Inoculation Department at St. Mary’s Hospital in Paddington, the Royal Institute of Public Health, Park Hospital in London, and the Lister Institute of Preventive Medicine–“where thousands of millions of microbes, known as the National Collection of Type Cultures, can be seen” (Anon. 1934).

Amongst these august institutions the Lister’s National Collection of Type Cultures (NCTC), described as a “menagerie of microbes” by *The Times* in 1925 (Anon 1925), has been conspicuously absent from historical accounts of microbiology and infectious disease in the 20th century. The same is true of other collections of its kind despite the number and scale of modern culture collections. The World Federation of Culture Collections, which acts as an umbrella body for culture collections internationally, lists some 861 culture collections, containing over four million deposited organism types.^1^ These are critical components of modern scientific infrastructure, yet we know almost nothing about the changing development, operation, and impact of these collections. For example, in her comprehensive account of microbes, food poisoning, and public health in Britain, Anne Hardy notes simply that “the history of [microbial] reference collections remains to be written” (Hardy 2015, p. 118). While historians are now beginning to turn their attention to these collections, detailed studies are still lacking. One notable recent exception is the account of two personal collections of phytopathogenic fungi and single-celled algae maintained by Johanna Westerdijk and Ernst Georg Pringsheim respectively. Exploring the interconnections between these two collections, and collectors, Charles A. Kollmer sets them against the activities of “other prominent culture collections such as the American Type Culture Collection […] or the National Type Culture Collection [*sic*]” (Kollmer 2022, p. 22). However, a full account of the NCTC, or any other national-level culture collection, has not yet been published. In this sense, this study of the NCTC serves as a template, highlighting how these more wide-ranging, more obviously institutional collections might be investigated in the network of microbiology.

Bruno Strasser’s recent and influential account of the emergence and role of datasets in the experimental life sciences provides a vantage point from which to begin a more systematic interrogation of culture collections on their own terms. Taking as the principal object of his study the microbial collections of the American Museum of Natural History prior to 1920, Strasser argues that this collection—forerunner to the American Type Culture Collection established formally in 1925—“illustrates particularly well the roots of stock collections in natural history museums and their similar trajectories” (Strasser 2019, p. 34). This, he contends, is reinforced by the view at the time “that microbes, like plants and animals, should be considered not just as pathogens of medical interest but as a part of nature” (Strasser 2019, p. 35). However, as I later show, the genesis and operation of the NCTC stands in marked contrast to the decidedly natural historical approach seen in the case of the United States; the NCTC was not merely characterized by “selecting strong cultures at the outset, providing suitable storage environments, and instituting safe handling procedures [which …] mirror historical (and contemporary) archive practices,” as was the case with the American collection (Sutherland 2023, p. 62).

On closer examination of the NCTC, several common preconceptions about its founding and operation—not least the claim that the focus in the early years of its operation was limited to human and other animal pathogens—do not stand up to scrutiny. In the first place, the collecting policy of the NCTC during its first two decades was profoundly broader. Only after the disruptive influence of the Second World War were the collections rationalized around bacteria of purely medical or veterinary relevance, with fungi, plant pathogens, and yeasts distributed elsewhere. The first decades of the 20th century witnessed the emergence of centralized collections of microbes, which circulated cultures internationally. These were frequently underpinned by necessary and significant sources of funding from both “wealthy industrialists and national scientific funding agencies” (Kollmer 2020, p. 22). Given Kollmer’s persuasive argument that microbiological research was “enriched” by late-19th and early-20th century technologies of production, a comprehensive understanding of the origins, functions, and impact of culture collections—which were fundamental sites of technique, classification, and practice development—is therefore critical (Kollmer 2020, p. 23).

It is important to emphasize that the NCTC was not the first culture collection, nor the first to share type cultures with other research institutions. However, as a direct consequence of World War One, the majority of earlier such collections were either prevented from operating, or had their collections dispersed or destroyed. Both the Pasteur Institute, based primarily in Paris but with an international network equally as broad as NCTC, and the Kral Institute in Prague, later relocated to Vienna (then Chicago, by which time most of the cultures had died), predated the NCTC, and each served as important nodes in more informal transnational culture exchange (Strasser 2019, p. 37). The American Type Culture Collection explored by Strasser, while not established formally until 1925, had its origins in the culture collections of the American Museum of Natural History, which were already widely known by 1920.^2^

In an era following significant changes in bacteriological research (Tomes 1999; Worboys 2000; 2007; Homei and Worboys 2013), these collections were not simply suppliers of authenticated and standardized microbial strains (though this was an important aspect of their function). Within just a few years of the NCTC’s founding it had also become crucial in shaping the focus of the discipline, carrying out independent and original research, particularly in bacterial systematics, and embedding the concept of standardization itself at the centre of modern biological inquiry. Its first curator—a term to be discussed further below—in post for almost a quarter of a century, played a pivotal role in developing the first international bacteriological code of nomenclature, attempting to reach international agreement on how to standardized aspects of the microbial world. The focus of the bacterial collections changed significantly over time, reflecting the shifting pressures and priorities of microbiology and medical science. Perhaps of greatest consequence, as I argue, the NCTC distinctively reflected and amplified existing transnational networks of imperial power, collecting organisms from and sharing them through localities strongly connected to the British Empire. At the same time as the Imperial Mycological Institute, also founded in 1920, operated from the Royal Botany Gardens in Kew, the selection and distribution of microscopic organisms for research purposes exerted powerful influence over what living beings, diseases and industrial processes were favored for inclusion, how they were studied, and by whom. The labelling of one institution as “imperial” and the other as “national” is incidental: each acted as a critical vehicle for reinforcing and expanding British scientific influence overseas.

The period in question, from 1916 to 1922, is short, yet it covers the NCTC’s planning, founding, and early operation, culminating in the publication of the first full catalog of the collection. Established originally as a collection of microbes of general scientific interest and importance, including yeasts and protozoa, the focus shifted only in 1947, after which NCTC concentrated on strains of medical and veterinary relevance (Holmes 2018; Public Health England 2015). Beyond this period the NCTC continued to play a pivotal role in major shifts in the discipline, leading the UK National Committee of the Commonwealth (formerly Imperial) Collections of Microorganisms and acting as a key institution within the International Committee on Bacteriological Nomenclature. During the second half of the 20th century these organizations were responsible for supporting and shaping key research shifts within the biological sciences, including the elucidation of the role of mobile genetic elements such as plasmids and bacteriophage on the evolution of bacteria (Fazal et al. 2019), major reclassification of bacterial strains on the basis of genetic material, and, from the 1980s, the harmonization of the nomenclature applied to bacterial species and genera through phylogenetics (Oren 2024). However, before historians can grapple with these highly consequential impacts of culture collections on bioscience, we must first turn our attention to the origins of the earliest still functioning example of their kind: the NCTC.

This article is structured in three sections. First, I explore the planning, founding, and extraordinarily rapid expansion of a major culture collection as a necessity for microbiology in Britain. Second, I uncover and account for the nature of early circulation and depositing of microbes, drawn principally from existing imperial scientific networks. Finally, I examine the complex discussions and disagreements about bacterial nomenclature and classification through the compilation and publication of its first catalog in 1922. This interrogation is supported by previously unstudied, and in many cases uncataloged, early records of the NCTC, including correspondence, catalogs, and minutes of various MRC committees tasked with the establishment, maintenance, and reimagination of the NCTC’s operations.

## Rationale and Foundation

In the first portion of this section I present a reconstruction of the specific exchanges, principally in the second half of 1919, which led to the NCTC being rapidly established. Events are detailed to make clear the various institutional and individual imperatives at work, providing the first evidence-based account of who was involved in establishing the form, functions, and institutional basis of the collection. In the second portion I will place it in the context of other contemporary institutions in order to consider exactly what *kind* of institution the NCTC was, and the ways in which it relates to other projects in imperial science.

At the very height of World War One, the then Prime Minister, Herbert Asquith, established in mid-1916 a Reconstruction Committee from within his own coalition Cabinet.^3^ It was tasked with considering programs of national reconstruction across nearly the full range of government activity, and sought input to develop a strategy to be implemented when the conflict ended. Among the many organizations consulted on this matter was the Medical Research Committee (MRC), established in 1913 primarily to oversee the distribution of funds arising from the passing of the 1911 National Insurance Act (Landsborough Thomson 1987).^4^ At a meeting on October 16, 1916, the Reconstruction Committee approached the MRC directly, with requests for suggestions as to “the direction of future policy in medical questions of public interest.”^5^ At subsequent meetings the MRC “considered, amended and finally approved” a memorandum with an expansive vision for post-war medical research and capability.^6^ Amongst the proposals outlined in this submission to the Reconstruction Committee, recalled later by Walter Fletcher, was the necessity of establishing a “National Institute for Biological Standardisation, in which a collection of standard cultures was urged as a necessary part.”^7^

As the first Secretary of the MRC, Fletcher (1873–1933) was instrumental in directing resources towards fundamental biomedical and biological research during the early years of the MRC’s operation, including major investigations such as those into nascent vitamin science (Anon. 1933). Yet the impetus to work towards the creation of a national collection of bacterial specimens to support research came not just from within the MRC, as has been previously suggested. In fact, despite the submission of the MRC’s memorandum to the Reconstruction Committee in 1916 the idea must have languished because it was not until some three years later, by the pathologist A. E. Boycott (1877–1938), that it was picked up. Representing the Pathological Society of Great Britain and Ireland, within which he was then a key figure as Secretary, and holding the editorship of the *Journal of Pathology* for  twelve years, he wrote to biologist C. J. Martin (1866–1955), Director of the Lister Institute, on July 26, 1919 (Martin 1939). Boycott—who had spent a highly productive three years based at the Lister from 1904 and had wide-ranging interests across the biological sciences—noted that the committee of the Pathological Society “had some discussion at their meeting yesterday about the establishment and maintenance of a collection of cultures of bacteria and possibly also protozoa” on the basis “that some source of standard cultures of known origin and history is an urgent necessity in this country” following the lack of availability of the Kral and Pasteur collections.^8^

Whether or not this suggestion was inspired by the continued loss of access to European culture collections as a result of wartime, or trans-Atlantic jealousy following the formal establishment of a culture collection at the American Natural History Museum in January 1911, is hard to determine.^9^ Whatever the circumstances, the intervention of the Pathological Society acted as a catalyst for the formation of the NCTC. On the back of the suggestion, Martin approached Walter Fletcher in September 1919 about a “bact[eria]l herbarium scheme.”^10^ Martin’s choice of language—describing the enterprise as a herbarium—reveals a desire, mirrored in other writings of the time from US bacteriologists such as R. E. Buchanan and David Bergey, to establish bacteriology on as sure a footing as disciplines such as botany, for whom herbaria had long-since been fundamental tools and sites of research. Regardless, Fletcher was hugely supportive of the suggestion and encouraged further by securing the backing of bacteriologist William Bulloch, who was a member of both the MRC and the Lister Institute Governing Body. The MRC, Fletcher noted, writing to Martin with a lengthy, full-fledged proposal, had already “put this forward as an urgent need.”^11^ Even though their preference was to integrate a collection of bacterial cultures within a broader “National Institute of Biological Standardisation, analogous to the National Physical Laboratory,” they were not prepared to delay; the MRC considered that “immediate arrangements should be made for a standard bacterial collection, without waiting for the realisation of the full scheme just mentioned.”^12^ The Governing Body of the Lister Institute, Martin confirmed, was similarly “alive to the necessity for a herbarium & sympathetic towards co-operation with the M.R.C. towards the formation of a representative collection” since they did not have the financial means to support such an endeavour.^13^

The full proposal, developed principally by Fletcher, envisioned an expansion of the small culture supply services already offered more informally by the Lister Institute, which was now to be augmented by support from the Medical Research Fund.^14^ As Fletcher made clear in the proposal, the MRC was at the time struggling to consider requests for new projects until “their own future and financial position were settled;” the plans for the new “herbarium” were therefore necessarily of a smaller scale, and built on existing arrangements.^15^ In this spirit, the options outlined by Fletcher might at first glance appear virtually indistinguishable from each other: either to provide funding and defer the responsibility of running to collection to staff at the Lister Institute, or to maintain “direct responsibility for this work,” with the collection only temporarily located at the Lister.^16^ However, Fletcher himself was alive to the consequences of each, cautioning Martin that were “the [Medical Research] Committee [to] retain responsibility for it, it would be subject to the disadvantages often attached to a bureaucracy.” Perhaps of more significance, Fletcher expressed his anxiety that “a feeling might arise, say, in Scotland or Ireland, that they had no effective influence” on the arrangements if the Lister Institute had complete control over the collection from the outset.^17^ This consideration Fletcher urged Martin “to be kept in mind” during discussions about practical arrangements for housing the collection, despite the fact that “at the moment this reflection seems academic.”^18^

Through a rapid exchange of letters the following week, Martin reassured Fletcher of the Lister Institute’s commitment to this “Bact[erial] Herbarium […] to the best of its ability.”^19^ Anxious to make progress, Martin requested to be kept informed so that “the matter could be brought before the G[overning] B[ody of the Lister Institute] on Dec 10th.”^20^ The formal proposal duly came before the MRC meeting on November 28, where the committee “agreed in principle [to] assume responsibility for a national collection of type cultures of bacteria and of other protista.”^21^ The presence of the taxonomic kingdom protista, which at the time included a broad range of unicellular organisms, and emerged from the wide-ranging 19th-century category “infusoria,” confirms that the original scope of the collection was not restricted to bacteria, and certainly not to those of a pathogenic nature.^22^

With agreement on an annual expenditure of £1000, the MRC empowered Fletcher to enter discussions to formalize the arrangements with a view to supporting the venture for an initial period of three years. Fletcher began in earnest, writing to Martin on December 3 to suggest terms of the arrangement. Since funding originated ultimately from the government, the collection would be “the property of the nation,” regardless of the role of the Lister Institute in managing its operation.^23^ Fletcher also put forward a proposal for the Lister Institute’s Chief Bacteriologist, John Ledingham (1875–1944), to maintain “general supervision of the collection,” with further staffing to include “at least one bacteriological assistant and a laboratory attendant.”^24^ Ledingham was at the time a widely-respected figure within British microbiology, having worked at the Lister Institute since 1909 (Clegg and O’Neill 2004).^25^ Fletcher expressed the hope that appointing someone of Ledingham’s stature would confer on the collection “that confidence to be felt by workers […] which would be necessary for its success at the outset.”^26^

Within a fortnight Martin had brought the proposal before the Lister’s Governing Body, where it was “cordially approved” by the group.^27^Martin confirmed that Ledingham would be responsible for selecting the “bacteriologist and his assistant,” and the process went swiftly; by January 7, 1920, Ledingham had taken over direct communications with Fletcher and introduced “Dr [Ralph St. John-] Brooks as Curator with Miss Mabel Rhodes as Assistant Curator.”^28^ Although Ledingham subsequently retained significant autonomy in the administration of the NCTC, initial decisions were informed directly by Martin. For example, as Ledingham wrote, “we propose to charge one shilling per tube [of requested cultures] as you suggested and as I think is only right with public money. Of course donors to the National Collection will be preferentially treated.”^29^ With these principles and personnel established, Ledingham advised to wait until operations were more firmly established before making “an announcement in the Medical Press (both British and Colonial).”^30^ British and colonial institutions and individuals were, therefore, privileged both in terms of access but also financially, being more likely to be aware of the potential to donate cultures of interest and benefit from reduced or waived costs in sourcing other cultures from the collection.

Ledingham himself drafted the provisional constitution and regulations of the NCTC following further discussions with Martin and Fletcher. These reinforced the original remit of the collection, which was to accommodate “bacterial cultures representing all departments of bacteriology (medical, veterinary and economic) received from workers all over the world, the intention being to form as complete a collection as possible of organisms of economic importance in the widest sense.”^31^ This ambition—to be comprehensive across the domain—presented challenges. In an additional note appended to the first draft of the document, Ledingham added one further clause to the original seven. As he wrote, “[i]n including organisms of industrial importance, it is recognized that special difficulties may be encountered arising both from lack of information of their peculiar bionomics + of the factors favouring their propagation in artificial culture.”^32^ This was complicated still further by the desire to “maintain certain protozoal organisms such as Trypanosomes which are constantly in demand for research purposes.”^33^

Ledingham sent forward the proposed constitution to Fletcher on January 21, 1920.^34^ Full confirmation of these arrangements was given by the MRC at their meeting just two days later, less than six months after A. E. Boycott had first written to Martin.^35^ Tellingly, perhaps, given that the scheme required formal ministerial approval, Ledingham’s cautionary note about the challenges of maintaining strains of industrial importance was not included in the final constitution.^36^ Fletcher accordingly wrote to Sir Robert Morant (1863–1920), the Permanent Secretary at the recently-created Ministry of Health, to advise him of the MRC’s intent to establish the collection (Fry 2009). In a revealing and significant departure from earlier discussions, Fletcher noted that “the central collection will serve as a standard of reference in work done at a great variety of centres in this country, while by proper interchange and comparison of types with other countries, a unity of standard can be maintained internationally.”^37^

Fletcher also claimed that the MRC were approaching the collaboration with the Lister Institute with a good deal more skepticism than he suggested elsewhere. While in correspondence with Martin and Ledingham he had been positive about the longer-term security afforded by committing to a minimum of three years’ support for the NCTC in the agreed form, he impressed upon Morant that the MRC had the “option of terminating this,” describing the plans as “an emergency arrangement […] with the Lister Institute definitely terminable at desire in two years” so that the activities of the NCTC might be integrated into the planned Central Institute (what later became the National Institute for Medical Research).^38^

The Minister of Health at the time—indeed, the architect of the Ministry of Health itself as successor to the Local Government Board in 1919—was Christopher Addison (1869–1951). Addison was not only a former physician and Professor of Anatomy at the University of Sheffield, but had also taken on the role of Minister of Reconstruction from 1917, with particular responsibility for postwar planning (Cooter 2004; Morgan 2011). It is therefore not difficult to imagine Addison’s enthusiasm for the planned enterprise, and Fletcher was able to write to Ledingham confirming ministerial approval just six days after his letter to Morant.^39^ The following day Fletcher wrote to both Ralph St. John-Brooks and Mabel Rhodes to formally offer them positions as Curator and Assistant Curator respectively, noting in his letter to St. John-Brooks that there was every prospect “of making useful and original inquiries” alongside regular duties in managing and maintaining the collection.^40^

The rapid establishment of the NCTC was enabled by the receptive context present across all three contributing organizations. First, the Pathological Society of Great Britain and Ireland, whose role was later excised from historical accounts of the NCTC’s formation, made visible the need amongst a broad community of researchers. Second, the MRC itself already had in view the prospect of creating a National Institute for Biological Standardisation, of which a culture collection was to be a key component. Finally, the Lister Institute itself had established practices of issuing culture samples from its own collection on an informal basis.

To complement the detailed microhistory of the interactions leading to the establishment of the NCTC outlined above, I move now to consider for a moment what *kind* of institution it was, before examining features of its operation.

In her wide-ranging and detailed account of how imperial endeavours in science manifested in colonial Africa, Helen Tilley argued that from the late 19th century onwards the sub-Saharan portion of the continent became “an imperial laboratory where political, economic, and scientific experiments could be pursued with relative impunity” (Tilley 2011, p. 313). In this context, an important driver was “the imperial imperative to *localize* knowledge,” that is to subject specific geographies— “soils, deserts, forests, diseases, climate, species” being some examples—to interrogation on scientific terms (Tilley 2011, p. 318).

In light of this, we can see the NCTC as an attempt to reify microorganisms, in the form of a pure culture, whilst preserving vital information about their provenance. This represented a quite different approach from other strategies aimed at mastering either microbes or environments. Unlike the exporting of bacteriological practices to distant laboratory settings (such as that seen in 19th century British India) which required the adaptation of existing methods to local environment and culture (Chakrabarti 2012), Fletcher, Ledingham and others were attempting to bring samples taken from colonial and other international settings into a space which was more readily controllable. To some extent, this represents an approach similar to field collecting practices of natural historians in preceding centuries: creating a contextualized collection such as those housed in museums for the purposes of preservation, classification, and ordering. The critical difference, of course, is that the focus of the NCTC was on distribution, and included a need to maintain the organisms they housed in a viable state, whether living or, later, in suspended animation.

All three figures most closely connected with the founding of the NCTC were concerned primarily with medicine and pathology. This was reinforced by the appointment of St. John-Brooks, with his mixed background as a medical scientist (Allen 1966). As we shall see in the following section, the collection’s remit rapidly expanded to include organisms of industrial importance—especially yeasts—as well as pathogenic fungi. How did this occur? In the case of pathogenic fungi, collaboration with the British Mycological Society was crucial; the Society appointed a committee of their own devising, including St. John-Brooks, to “consider and advise upon the ways in which the Collection may be made valuable to mycologists and to the study of fungi” (Medical Research Council 1922, p. 4). To lessen the burden on St. John-Brooks and Mabel Rhodes, a duplicate set of all the fungal type specimens was maintained in “the Botanical Department of the British Museum (Natural History)” (Medical Research Council 1922, p. 5). While the NCTC was formally based within the Lister Institute, therefore, we can instead see how its expertise was somewhat more distributed in practice, reflecting its more diverse initial collection strategy.

As far as industrial strains were concerned, the Lister Institute had rapidly become a focus for research-industry collaborations. As long-time Lister staff member Harriette Chick noted, wartime:

revealed the necessity for research in many fields, and particularly in the chemical and pharmaceutical industries […] At various times the Medical Research Council, the Empire Marketing Board, the Department of Scientific and Industrial Research [formed in 1915], and the Ministry of Agriculture provided for specific projects the salaries of senior workers, who usually became honorary members of the staff. (Chick 1971, p. 127)

In fact, Nobel Prize recipient and long-standing Lister Institute (1907 to 1930) biochemist Arthur Harden (1865–1940) already had a personal collection of yeasts which were integrated into the NCTC holdings as well as others from the likes of Leeds-born UCL chemist and frequent brewery collaborator Alfred Chaston Chapman (1869–1932) (noted in both Chick et al. 1971, p. 141, and Medical Research Council 1922, p. 35).^41^ Indeed, Chapman himself contributed strains of a wide range of organisms of industrial relevance, including *Torula* (of potential practical use, but also pathogenic), *Schizosaccharomyces pombé* (“discovered by Saar in ‘pombé’ [African millet beer]”), and *Pichia farinose* (“discovered in Danzig ‘Jopen’ beer”) (Medical Research Council 1922, pp. 27, 31, and 33). The Institute’s early work on the investigation of antitoxins and antisera were also fundamental in establishing connections with the pharmaceutical industry. While it is less clear that there were specific overtures from the NCTC to industry about the collection of distribution of strains, it is not a stretch to attribute the rapid engagement with commercial entities in a range of industries to existing relationships. We know that the culture collections at the Lister were small but “internationally known to those engaged in the classification of microorganisms and in medical and bacteriological research” (Chick et al. 1971, p. 141).

The NCTC therefore embodied a distinctive constellation of features of imperial microbiology, some of which were common to laboratory spaces in Britain and elsewhere, others less so. Explicitly framed by the MRC as an exercise in post-war rebuilding, it drew together in the Lister Institute biological samples from throughout the British Empire, and was focused on organisms of medical and industrial relevance to British authorities. In the following section, I examine which strains were collected, from where, how, and to where these were distributed, and some of the tools and techniques used by NCTC staff to ensure the reliability of the strains in their care.

## Early Ways of Working and Networks

Since we know that for most of its early operation the NCTC rested on existing networks and connections, the background of the curatorial staff warrants some exposition. A native of Dublin, the first Curator, Ralph St. John-Brooks, studied Natural Sciences at Trinity College in the city, graduating in 1904, and then completed medical study, also in Dublin. After this and in common with many of his contemporaries, he spent the bulk of the next decade overseas and in military service, first in the West Indies as “special sanitary investigator” in the Windward and Leeward Islands (1913–1914), then as Secretary and Investigator to the Commission for Plague Investigation in India (1914–1915). He then joined the Royal Army Medical Corps (RAMC) as a bacteriological specialist, working first at the County of London War Hospital (1915–1919) and then at the Royal Army Medical College from 1919, where he came to the attention of Ledingham (Allen 1966).^42^ It was St. John-Brooks’ connections through the RAMC which formed the basis of many early additions to the incipient collections of the NCTC: he later recalled how he “added to these by collecting cultures from personal acquaintances whom he visited in his off-duty hours,” since he initially retained formal connection with the RAMC before his demobilization in March 1920 (Allen 1966, p. 165; Anon. 1920b).^43^

While there are more extensive records of St. John-Brooks activities during his tenure as Curator, several sources attest to Mabel Rhodes’ vital role as Assistant Curator. Privately educated, and seemingly without having attended university, she spent her entire professional career at the Lister Institute, as assistant first (1907 to 1915) to protozoologist E. G. Minchin and then (1915 to 1919) to the hugely influential experimental pathologist and nutrition scientist Harriette Chick (1875–1977) (Ainsworth 1996, p. 140).^44^ From the outset Ledingham, as Director, regarded Rhodes as at least equally important as St. John-Brooks in the smooth functioning of operations, writing to Fletcher in March 1922 to request a salary increase for them since “both worked hard to make the venture the success” that it was.^45^ Shortly after, Rhodes and St. John-Brooks co-authored an article exploring the properties of organisms responsible for causing fowl typhoid, demonstrating Rhodes’ crucial involvement in early research activities with the collection (St. John-Brooks and Rhodes 1923). This is further reflected in the fact that when John-Brooks was absent owing to illness for a period of some eighteen months during 1926–1927, Rhodes “had entire charge of the Collection” (Lapage c.1971, p. 4). Rhodes’ publications during her time at the NCTC included papers on typology and, most notably, culture preservation and management (Rhodes 1950; Rhodes and Fisher 1950; Felix and Rhodes 1931). She remained until November 1949—outlasting St. John-Brooks—and in 1948 the incoming Curator, Samuel Tertius (S. T.) Cowan (1905–1976) recorded her comprehensive knowledge of the collection in his first impressions on arrival: “the number [of a desired strain] was found either in Miss Rhodes[’] head or in a curious book called ‘The Active List[’].”^46^

An important aspect of the collection’s early operation was maintaining records of cultures both received and dispatched.^47^ These show that the first subcultures–which included *Shigella flexneri, Salmonella paratyphi-B*, *Bacillus dysenteriae* and *Bacillus typhosus*–were issued on January 7, 1920 to the Medical Superintendent at the Graylingwell Mental Hospital (formerly the West Sussex County Asylum) in Chichester, a role then held by Harold Kidd.^48^ Like many other asylum superintendents at the time, Kidd was an active researcher, and had brought Alexander Fleming, also an early recipient of NCTC strains, to the asylum to support his own efforts to explore general paralysis of the insane (Wright 2016). Given the timing of the request, the subcultures issued to Kidd (NCTC strains 3, 6, 11, 12, 13 and 14) were almost certainly from within the existing Lister Institute’s collection.^49^

Within the first year, Rhodes and St. John Brooks had sent cultures as far afield as Australia, India, Uganda, South Africa, New Zealand, Sierra Leone, Peru, and Malaysia, and to prominent commercial entities such as Boots, Allen & Hanbury’s and the Jeyes Sanitary Compounds Company. Two samples of *Salmonella* went to the Lever Brothers in Port Sunlight on January 17, 1921 at a total cost of two shillings, while no fewer than thirty-one subcultures were sent *en masse* to the Pasteur Institute of Burma in Rangoon just a few days later. On February 22, 1921 several different strains of *Staphylococcus aureus* were dispatched to Allen & Hanbury’s.^50^

Information about the provenance of many, but not all of these early strains still survives. For example, the specific strain of *Lactobacillus bulgaricus*, a gut-dwelling organism found in yogurt, listed as number 76B was deposited by John Eyre on December 6, 1920. Eyre, who was Professor of Bacteriology at Guy’s Hospital, affiliated with the University of London, obtained the sample from food manufacturers Aplin & Barrett in around 1917. More than this, Eyre noted that “[i]t has been growing in the Laboratory for the past twelve years. I originally got it from Paris, from a little Laboratory that Metchnikoff himself established.”^51^ A subculture of this same strain was sent to Allen & Hanbury’s in August 1921.^52^

Through the course of the first month of the NCTC’s operation in January 1920, a total of sixty-one strains were issued to fourteen different researchers and organizations. Given that the NCTC had not yet been formally announced to the profession, those requesting strains must either have already known about the informal collections service provided by the Lister Institute, or been closely associated with individuals and institutions involved in the establishment of the NCTC, or both. This is confirmed by the records of those who requested and received strains at this time. Three of the fourteen sets of initial requests went to other figures at St. John-Brooks’ own institution—the Royal Army Medical College at Millbank—while two samples sent to Burroughs Wellcome and another destined for the Municipal Health Department in Shanghai demonstrate the reach of the collection to industry and internationally from the outset.^53^ Others went to hospitals in Bristol, London and York, as well as the Health Department in Ayr, Scotland. The pattern of distribution of organisms during the early months remained largely unchanged. The Royal Army Medical College was the most frequent recipient, while other commercial entities such as Allen & Hanbury’s and the Boots Pure Drug Company also requested several sub-cultures.^54^

In April 1920, less than ten months after the first contact between members of the Pathological Society of Great Britain and Ireland and the Lister Institute, the MRC arranged to promote the NCTC to professional communities through a circular in the medical and scientific press. Fletcher shared a draft of this with other MRC members, including William Bulloch and Henry Dale, including a list of intended recipients.^55^ This was issued rapidly to British publications: both *The Lancet* and *British Medical Journal* carried identical articles on May 15, 1920 announcing the establishment of the NCTC to “collect and maintain bacterial and protozoal strains of medical, veterinary, and economic importance,” with an initial emphasis on “fully authenticated strains of pathogenic organisms” (Anon. 1920a, p. 1081; Anon. 1920b, p. 682).^56^ The article is noteworthy for several reasons. First, it acted as a call to researchers and “earnestly invited” strains for inclusion within the collection, preferably “accompanied by the fullest particulars as to source and date of isolation and, if possible, by clinical and epidemiological notes” (Anon. 1920a, p. 1081). Second, the decision about whether to accession such strains was ultimately “left to the discretion of the director;” strains had to meet a presumed threshold of “sufficient importance” (Anon. 1920a, p. 1081). This indicated clearly that novelty alone was not a qualifying criterion for inclusion of a strain in the collection, and that the priorities of Ledingham—and, by extension the Lister Institute and British scientific establishment—determined the nature of the organisms kept and made available. Finally, the intention was to prepare and circulate a catalog outlining both the list of strains available and details of their origin.

In the weeks that followed, we see further how the original activities and reach of the NCTC mirrored British imperial networks. In addition to British-based periodicals, such as the newly-established *Journal of Experimental Pathology*, the MRC also sought the guidance of St. John-Brooks about where to announce the collection. His suggestions were limited to a list of English language medical periodicals in Australia, Canada, India, New Zealand, and South Africa, as well as the *Journal of the Royal Army Medical Corps* and *Journal of the Royal Naval Medical Service*.^57^ Landsborough Thomson duly issued these on behalf of the MRC on May 31, 1920, thereby largely limiting the initial visibility of the NCTC’s activities to medical networks within the British Empire.^58^

In July 1920, Fletcher requested from Ledingham an update on the work of the NCTC for inclusion in the MRC’s annual report.^59^ The report, most likely generated by a combination of Ledingham, St. John-Brooks, and Rhodes, indicates the scale of culture acquisition and circulation. The collection had already grown to over 800, and the same number having been dispatched.^60^ The assertion in the report that the collection had achieved significant international reach, both within and beyond the British Empire, is confirmed by dispatch records. Even by the end of March 1920, for example, sub-cultures had been sent to researchers and institutions in Baghdad, Copenhagen, Valetta, Johannesburg, Bombay, New Haven, and Leuven.^61^

By the end of the first year of the NCTC’s operation, between 1,800 and 1,850 individual sub-cultures had been issued.^62^ This we can corroborate fairly precisely from the contents of the original dispatch box. However, in contrast to this, it is somewhat more unclear exactly which strains were deposited, when, and by whom. St. John-Brooks’ later reflections on the NCTC’s first twenty years of operation suggest that several figures—Muriel Robertson, Arthur Handen, Frederick Andrewes, A. Stanley Griffith, S. R. Douglas, A. Klöcker, John McFadyean, Charles Thom and A. C. Thaysen—were especially prominent amongst the donors of cultures.^63^ What we do know is that by April 1920 the scale of the collection had grown such that *newly-acquired* cultures, not just those that were already in the Lister collection, were being circulated to recipients, including six NCTC strains numbered between 338 and 422, sent to the Royal Army Medical College.^64^ Given that multiple sources point to the original Lister Institute collection comprising no more than 200 cultures, these must have been acquired in the early years of 1920.

According to Mabel Rhodes’ own account, the initial laboratory for the NCTC in the Lister Institute was “one of the largest and most pleasant in the building,” but poorly equipped, necessitating “scrounging and borrowing from other departments” (Rhodes 1950, p. 1). The practical methods of maintaining and generating cultures relied largely in repeated sub-culturing. This presented several challenges to St. John-Brooks and Rhodes. In the first place, aiming towards a maximally “pure” version of each culture demanded that they be kept meticulously apart: a problem as the collection rapidly increased in size. It also constituted a significant hazard, since the method occasionally required the passage of more fastidious organisms through animals. This resulted, in 1923, in both St. John-Brooks and Rhodes being the first people in Britain to contract tularaemia—the causative organisms of which they were passing through a guinea pig—and becoming seriously ill, along with long-time Lister bacteriologist Dr Harry Schütze. Ledingham and Fletcher exchanged anxious letters about the incident, attracted significant press attention, and resulted in the abandonment of any further use of animals (Anon. 1923).^65^

In order to maintain as closely as possible the “integrity” or purity of the strains, they experimented with various methods for sealing cultures, including paraffin wax, “Playwax,” gutta-percha, and “Plastocene.”^66^ They used, but also furthered, established methods of microbial cultivation, relying on plating and slopes, with existing media such as MacConky’s agar most frequently pressed into service. The reliance on techniques widely-known in the field had the advantage of making any refinements readily accessible to a wider pool of microbiologists and other users of the collection, who were required to engage in specific laboratory practices to ensure the continued viability of samples issued to them. The early publications by St. John-Brooks and Rhodes, as well as well-documented research activity conducted by Australian virologist and phage biologist Frank Macfarlane Burnet during his time at the NCTC deputizing for St. John-Brooks in 1926, attest to the organization being concerned with active research as well as maintaining and expanding the collection (Sankaran 2010; Sexton 1999, p. 50).

Rhodes and St. John-Brooks also had to balance the needs of the organisms, frequently relying on trial and error with different forms of equipment. Of these, the emblem of pure culture—the Petri dish and its solid growth media—was just a single, albeit near-constant, component, highlighting the variety of methods and tools required to cultivate pure cultures of a huge range of microorganisms, each requiring subtly different conditions for (optimal) growth. The continued refinement of culture methods was a necessity at the outset of the NCTC’s operation. Given the reliance on these, it is therefore somewhat surprising that experimentation with methods in freeze-drying—widely known following the publication of American microbiologist Homer Smith’s findings in 1921—does not appear to have been part of the approach at the NCTC until well over a decade after its establishment (Smith 1921). Indeed, according to a later account by the third Curator, Stephen P. Lapage (in post from 1965–1978):

[in 1933] a few experiments were carried out in freeze-drying using the method of Swift in which the cultures are immersed in a freezing mixture during the process of desiccation. This method was abandoned in favour of the technique described to Dr St John Brooks by Professor Sordelli in 1934 during a visit from Buenos Aires. The method involves freeze-drying the cultures over phosphorous pentoxide and has been described by Rhodes (1949, 1950). It was adopted in the NCTC from 1934 onwards.^67^

The scope of the NCTC’s collection was therefore determined as much by the technical capacity and methods of culturing and preservation as it was by existing networks and connections, its visibility amongst different institutions and regions, and the priorities of its staff. Even bacteriological manuals such as that issued in 1923 by the Society of American Bacteriologists, *Manual of Methods for Pure Culture Study of Bacteria*, were rendered rapidly out-of-date by the dramatic expansion of types of organism collected by the NCTC and, later, the ATCC. The innovations at the NCTC therefore do not sit neatly with Mathias Grote’s observation that, “if one compares the skills of pure culturing from the early decades of the 20th century to their practice a century later, the impression of continuity is striking” (Grote 2017, p. 13). While this may have been true of most laboratories—which were typically interested in a far more restricted range of organisms at any one time—large-scale, diverse culture collections which failed or succeed on their strength in maintaining purity of culture clearly needed to develop a far broader range of strategies for effective preservation and isolation of their charges. Indeed, as St. John-Brooks’ successor, S. T. Cowan, would discover upon inheriting a system seemingly unintelligible to the uninitiated, purity of record-keeping was at least as important as purity of the strains themselves.

Not all early users of the collection shared the zeal or necessity for such diligence in their own records. This makes the reconstruction of early patterns of use, distinct from distribution, more challenging. However, in closing this section with a particularly notable case study, I hope to illustrate that we can see the impact of the NCTC—albeit within the context of a particular individual—from the outset.

**Figure 1:**
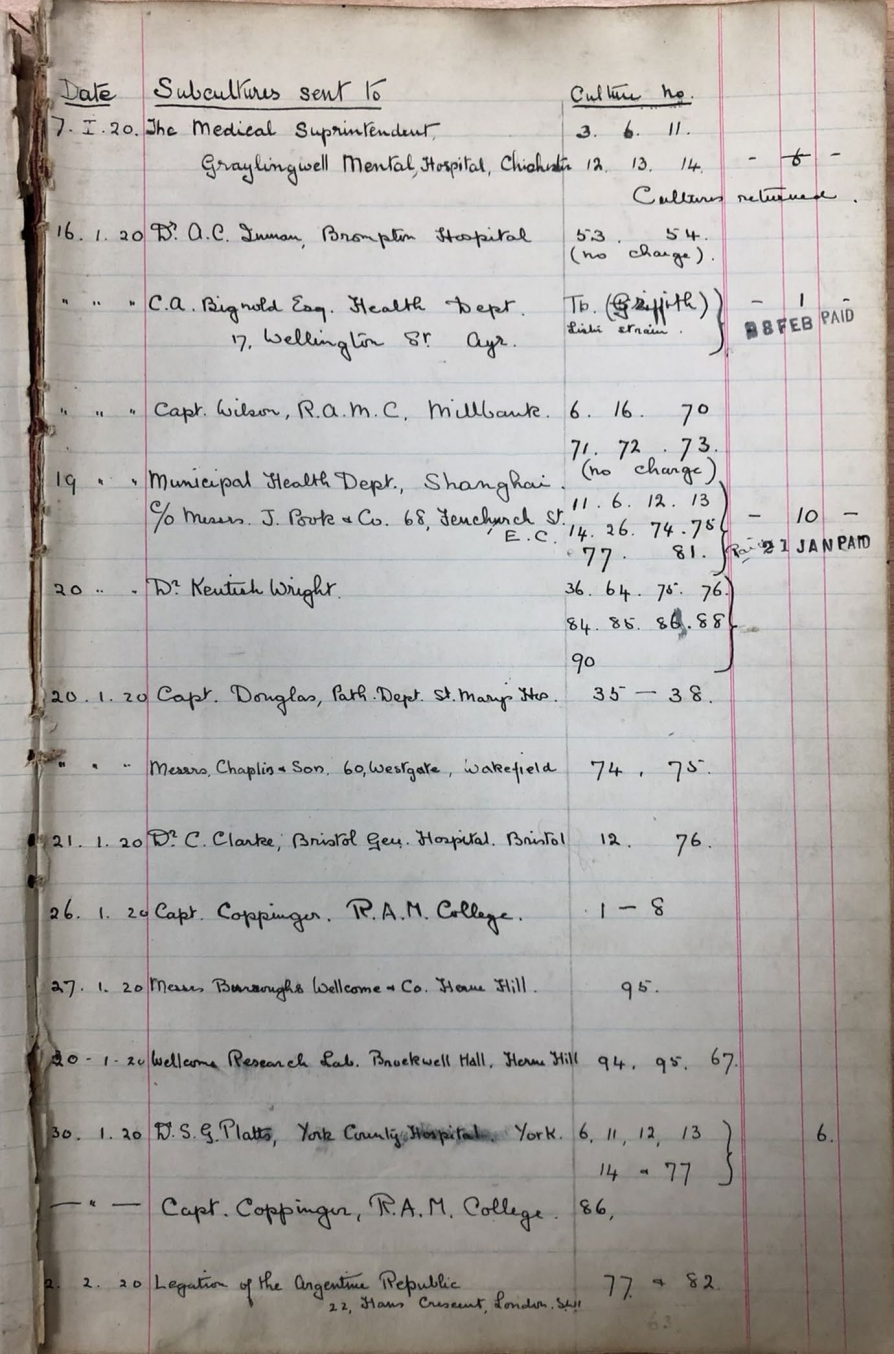
The first page of the Despatch Book No. 1, which included details of the recipients of NCTC strains from January 1920 to May 1923. (“Despatch Book No.1” (1920–1923), NCTC. Reproduced by kind permission of the UK Health Security Agency and National Collection of Type Cultures)

**Figure 2:**
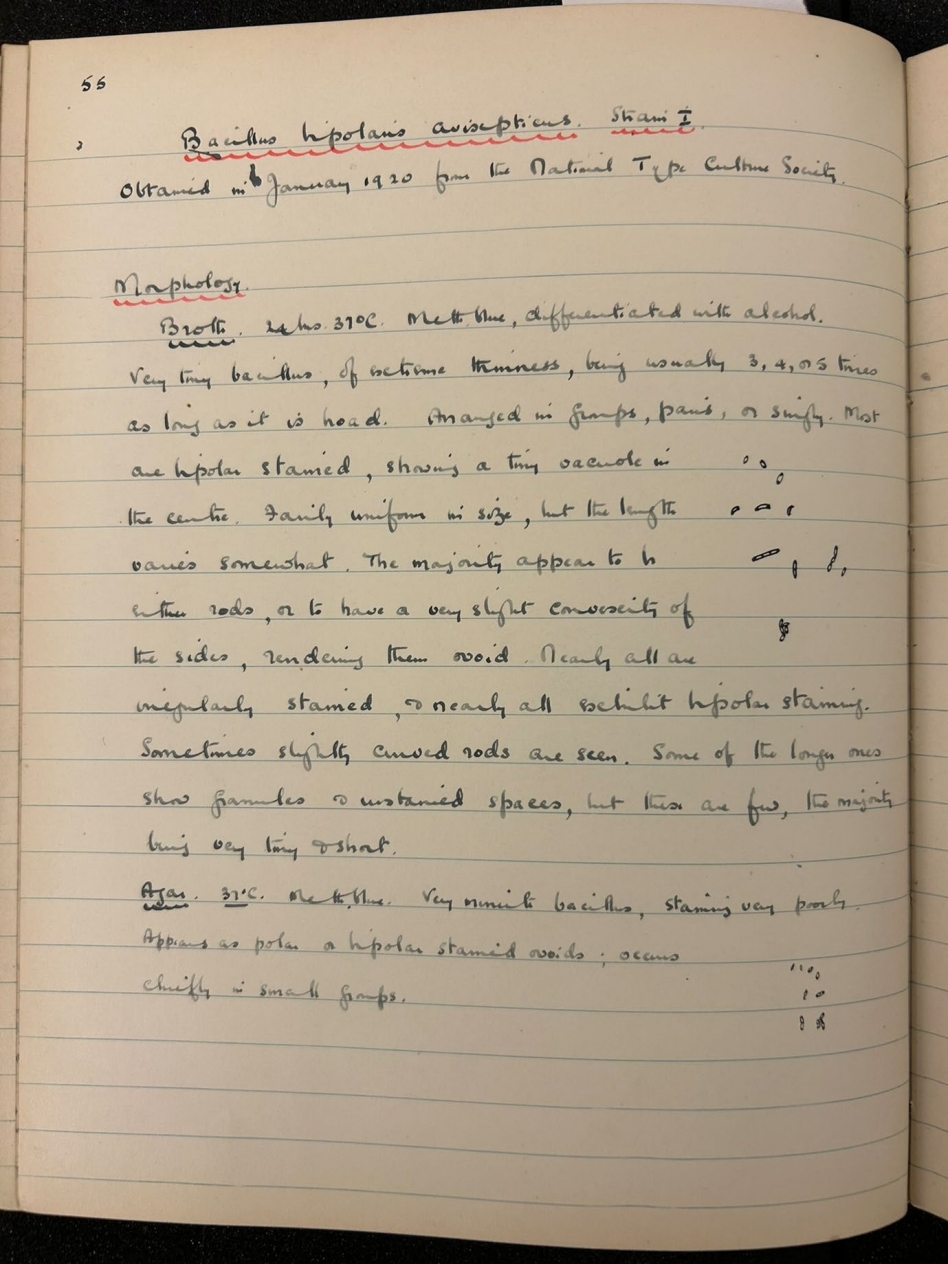
A page from G. S. Wilson’s notebook including a reference to the provenance of *Bacillus bipolaris avisepticus*, which Wilson received in January 1920. (Graham S. Wilson, “Notebook recording work on bacterial isolates: cultural characteristics, 1919, 1920, 1924, 1925, 1926,” 1920, p. 55, Wellcome Collection, PP/GSW/B/1. Reproduced by kind permission of the Wellcome Collection and Estate of G. S. Wilson)

As well as those listed above, one other early recipient of strains from the NCTC was medical bacteriologist Graham Selby Wilson (1895–1987), described in his obituary in the *Journal of Medical Microbiology* as “probably the most influential British microbiologist of the 20^th^ century” (Parkar 1988, p. 301). In 1919 and early 1920, Wilson was recently returned from military service and based initially at the Royal Army Medical College (RAMC). Shortly after this he secured a position at Charing Cross Hospital where he carried out experiments in culturing and bacterial growth (Anderson and Williams 1988, p. 890). While at the RAMC—where he was a colleague of St. John-Brooks—he was one of the earliest to request strains from the NCTC. On January 16, 1920, as part of just the second set of dispatches from the newly-formed NCTC, Wilson was sent a batch of six organisms, including *Bacillus avisepticus* (the causative organism of fowl cholera, now reclassified as *Pasteurella multocida*) and *B. dysenteriae* (Flexner) (see Figure 1).^68^ The ultimate origin of these was the collection at the American Museum of Natural History and the Pasteur Institute respectively, illustrating the strong likelihood that a significant number of strains of international origin were already part of the Lister’s own collection (Medical Research Council 1922, pp. 10 and 12). Wilson set to work with the strains, recording in his notebook their provenance (see Figure 2), though he mistakenly referred to both the “National Type Culture Society” and, elsewhere, the “National Type Culture Institute,” in his notes from 1920.^69^

His work with these organisms took several forms: cultural reactions on various growth media, such as on various agar and gelatin plates, in nutrient broth and peptone water, and on blood agar; observation of cellular morphology and characteristics; macroscopic colony morphology; and staining reactions. Following his move to the University of Manchester and then the London School of Hygiene and Tropical Medicine in 1923 and 1927 respectively, Wilson continued to employ and develop bacteriological techniques (Anderson and Williams 1988). His own notes recount how, for example, the examination of microbial strains from samples taken either during illness or post-mortem, in cases of everything from food poisoning to suspected anthrax infections, continued to be a feature of his work throughout the 1920s and 1930s.^70^

He must have been satisfied with the performance of his initial receipt too, for on June 15, 1920, a further seven strains were sent out to Wilson in his new location at Charing Cross Hospital.^71^ These included NCTC 90, a strain of *Bacillus paratyphosus C* originally isolated by Hirschfeld in Salonika and *Bacillus suipestifer* originating from the mesenteric gland of a monkey “that died in the course of a dietetic experiment” at the Lister Institute in 1917 (Hirschfeld 1919; details of the origins of *B. suipestifer* in Medical Research Council 1922, p. 18).

We know that in 1919–1920 Wilson was also working with a range of other organisms including *B. pseudotuberculosis* though it is unclear whether he sourced these from the NCTC’s collection. His notes, however, also provide definitive evidence of sources of cultures, beside the Lister Institute, prior to the founding of the NCTC. In December 1919, for example, he acquired a strain of *B. pestis* from the Royal Army Medical College.^72^ This confirms that—as might be expected—the NCTC initially served as one place amongst many from where strains might be obtained.

Further evidence confirms that Wilson’s connections with the NCTC continued for many years. In 1932, for example, he deposited a strain of *Brucella melitensis* which remains in the NCTC catalog to this day (UK Health Security Agency 2025a, b). Further deposits from Wilson that continue to be part of the collection include *Brucella suis* (NCTC 4490) and *Brucella abortus* (NCTC 4487), both accessioned in 1934, and *Bacillus subtilis* (NCTC 5398) in 1938.

The purpose of outlining this level of detail about Wilson’s early and continuing engagement with the NCTC is twofold. First, at the most general level it serves as a case in the early use of the NCTC, indicating that strains from the collection—alongside those from other sources such as the RAMC—were integrated into existing microbiological research. Second, and more specifically, it is not a stretch to imagine that while Wilson may have found other routes to both securing and sharing strains relevant to his and others’ research, the existence of what became a larger-scale reference facility facilitated access to, and knowledge about, a wider range of microorganisms.

Wilson was undoubtedly in a privileged position, and able therefore to make use of the collections right from the outset. Others were also in this position. However, the need for the NCTC to bring details of those strains which were available, and how they might be accessed, was an essential next step to increase the visibility, viability, and use of the collections.

## Towards a Catalog: Scope and Meanings

One of the original ambitions was the production and circulation of a catalog outlining both the cultures in the collection and some details of their properties and provenance. St. John-Brooks had been actively engaged in compiling the catalog from early in his tenure and shared draft material with Landsborough Thomson in June 1921.^73^ Far from this being a dispassionate list of the organisms available, it ignited fervent discussion. Landsborough Thomson immediately made several suggestions, noting that the headings for groups of organisms were confusing, some listed under “infections,” others under “disease” and still more under groupings which referenced the names of organisms themselves or “a branch of science.”^74^ He emphasized the importance of presentational clarity by invoking the views of Fletcher who, he noted, “considers the question of form and typography to be of great importance.”^75^

Landsborough Thomson then set about consulting more widely. He organized a meeting between William Bulloch, C. J. Martin, John Ledingham and Fletcher to discuss the matter further, and forwarded drafts of the catalog to Edgar Schuster, Head of the Publications Department at the National Institute of Medical Research for his input.^76^ Schuster’s feedback—sent directly to Fletcher—was scathing. After “discussing the system of classification […] with [protozoologist Clifford] Dobell […] it involves many quite unnecessary difficulties [… including] a most illogical arrangement of the organisms.”^77^

The alternative proposed by Schuster was simple: to dispense with St. John-Brooks’ plan for a catalog based on aetiology and instead arrange organisms alphabetically under headings of “Bacteria, Fungi and Protozoa.”^78^ Fletcher had heard enough, and he wrote to Ledingham to propose almost wholesale the system advocated by Schuster and Dobell. Fletcher explained that St. John-Brooks’ preference for a catalog based on aetiology was sure to “become out of date with advancing knowledge,” and suggested that the shortcomings of the draft lay in it simply being built on the Lister’s existing, informal, record of their culture collection.^79^

Ledingham duly passed on the letter to St. John-Brooks who was far from impressed. He argued that “the idea of a catalog which does not become out of date with advancing knowledge is a contradiction in terms.”^80^ He also pointed out that the supposedly neat classification under headings of bacteria, fungi and protozoa would not be possible. Revealingly, he held that “Bacteria (Fission Fungi and Schizomycetes) are themselves a class of […] Fungi.”^81^ Ledingham too compiled a response to Fletcher, and sent the two letters together. His, while still constituting a rebuttal on largely the same grounds as St. John-Brooks, was rather more diplomatic in tone. Nevertheless, he still argued that “the present state of knowledge as to classification and nomenclature militates against any serious attempt to carry out the alphabetical system [proposed by Schuster and Fletcher] in its entirety without falling foul of classification experts past present and future.”^82^

For his part, Fletcher was persuaded by the representations of Ledingham and St. John-Brooks and hoped that they could “proceed at once” to finalize the catalog.^83^ He also presented the letter to Bulloch and urged him that, “if Ledingham and his colleagues have strong feelings in one direction, we should accept their views if we can.”^84^ Bulloch was relaxed about the affair and could see that “there is a good deal to be said for Ledingham’s position.”^85^ Dobell and Schuster were not so easily persuaded. After a brief acknowledgment of Ledingham’s position, Schuster noted drily to Fletcher that Dobell wanted to meet in person “to dissect their letters verbally.”^86^ The two met on August 3, and Dobell laid out his counter-objections in detail the following day, responding to St. John-Brooks rather than Ledingham, perhaps at the suggestion of Fletcher for fear of disrupting the essential collaboration between the MRC and Lister Institute. Dobell reinforced his view “that an alphabetically arranged catalogue is capable of infinite expansion,” claimed that St. John-Brooks’ understanding of the blurring of the boundaries between bacteria and fungi “seems to me nonsensical,” and argued vehemently that “the catalogue should be a catalogue, and not a new kind of classification.”^87^ As he summarized his view:

The easiest system to follow, and the most stable, is the alphabetical. A subject catalogue can easily be added, and modified from time to time, as necessary. But to make an inconsistent and incongruous ‘etiological’ classification serve as the backbone of the catalogue seems to me the worst possible system to follow.^88^

Fletcher took some time to consider this before replying to Ledingham. When he did, he emphasized the need for the catalog to be constructed alphabetically, with a subject index “including aetiological findings” as a supplementary aid.^89^ Ledingham and St. John-Brooks were satisfied, and proceeded to provide Schuster with material for the index.^90^ This compromise resulted in a catalog which was shaped not only by knowledge of bacterial species and properties, but also the need to make this intelligible to the catalog’s intended audience, which included researchers from various biological specialisms and medical and public health practitioners.

Progress in the production of the inaugural catalog moved rapidly. By March 1922 the catalog was finished and circulated through selected avenues in the medical and scientific press. Responding to the publication, an article in *The Lancet* made explicit comparisons between the utility of the catalog and the critical foundational work in the maintenance of specimens in other scientific disciplines, including botany and zoology, noting that an “orderly arrangement of objects and an adequate nomenclature […] must form the basis of any further progress [… in] bacteriology” (Anon. 1922, p. 492). Perhaps reflecting the multiple voices at work in determining the structure of the catalog, *The Lancet* reported that the “arrangement of the catalog is illogical but eminently practical, and the authors are to be congratulated on having resisted the temptation to essay a classification” (Anon. 1922, p. 493).

As an early attempt to systematically present a collection of microorganisms to a mixed international audience, the catalog reveals the uncertainty about how the microbial world should, and could, be arranged. Indeed, the introduction to the catalog noted “the present […] problems involved in the classification of micro-organisms” (Medical Research Council 1922, p. 5). But it also highlights how the proclivities of a relatively small number of individuals—including science administrators as well as practicing scientists themselves—shaped its form. The inclusion of many strains originating from areas connected to the British Empire and theaters of war—such as *B. dysenteriae* direct from Flanders, Gallipoli, and various different military hospitals; several strains of *B. paratyphosus C* from Bagdad, and *B. pestis* from Bombay and Ceylon—as well as numerous strains of *Aspergillus* from the London Tube Railway, ensured that the representation of organisms in the collection mirrored their distribution within British spheres of interest (Medical Research Council 1922, pp. 12, 16–17).^91^ Further, the inclusion of kinds thought to be “interesting species” indicates the license which architects of the collection had in determining which organisms may or may not have been worthy of attention (Medical Research Council 1922, p. 4).^92^

This section has made visible for the first time the intensive debates which took place surrounding the content and form of the first catalog of the NCTC. Presenting these in detail has been a necessity and reveals the strength of feeling amongst key figures within the British medical establishment. In common with many similar publications emanating from the MRC during this period, the catalog carried a weighty sense of the British establishment, published by His Majesty’s Stationery Office, and purporting to be an authoritative, comprehensive, and accurate representation of microbial life. The fact that the final product was subject to such significant behind-the-scenes wrangling illustrates the lack of certainty about how to imagine relationships between microbial organisms, to say nothing of the fact that the organisms contained in them were more a reflection of expanded networks of British imperial microbiology than of a mythological, objective community of microbes. Indeed, as those associated with the NCTC would later find, even after the appearance of Bergey’s *Manual of Determinative Bacteriology* (Bergey 1923), an enterprise emanating from long-standing attempts within the Society of American Bacteriologists to provide a comprehensive finding aid and classification of microbial life, resistance to adopting this “American model” continued.^93^ Even as late as 1958, under the tenure of S. T. Cowan, the NCTC catalog was at pains to note that the “Nomenclature does not follow the usage of any one textbook of determinative bacteriology,” denoting the ongoing lack of consensus in bacteriological systematics (Medical Research Council 1958, p. 1).

## Conclusion

What does an account of the early years of the NCTC tell us about microbial collections and their custodians? In the first place, we see how, as one might expect, those tasked with leading such collections wielded outsized influence in establishing taxonomic standards and methods to be applied to the microbial world. In this regard St. John-Brooks, and by extension the NCTC, were especially active. Second, advocates sought to draw on the need for post-war rebuilding to establish or expand culture collections as a means of securing national prosperity and further international cooperation within science. Cultures collections were analogous to other national- and supra-national-level institutions, such as the UK National Physical Laboratory (established in 1900), which set out to establish agreed standards in other scientific domains.

The NCTC was established at a time when national infrastructures in science took on outsized importance. However, it also appeared at a time when the Pasteurian and Kochian models of bacteriological practice and organization which had characterized the late nineteenth century had all but dissolved. As noted by numerous intersecting historical accounts, by the early decades of the 20th century the microbial world was widely regarded as being far more complicated than had previously been reckoned (Hardy 2015; Wall 2013). Classification was at the heart of attempts to make this world intelligible. As Christoph Gradmann has noted, “[t]he construction of bacterial etiologies,” largely in the mode of Robert Koch, “led to a new understanding of infectious disease that was based on a combination of nosology and taxonomy” (Gradmann 2009, p. 68). Exercises in classification and identification were far from novel—Buchanan’s 1925 text of bacterial systematics recounts the precise details of numerous prior taxonomies in eye-watering detail across nearly a hundred pages—but they continued to be a major preoccupation of microbiologists in the interwar period when practical aspects of bacteriology such as the development and testing of a wide range of bactericidal substances seemed to rest on a more comprehensive awareness of the different properties, features, and behaviors of microorganisms (Buchanan 1925, pp. 15–108; Méthot 2016).^94^ In this way, we can see the motivation behind the NCTC, and its early activities, as reflective of the challenges in the field of microbiology as well as an attempt to establish robust forms of scientific infrastructure within imperial British networks.

However, it was quite different in character to the failed transnational exercise of imperial power embodied in the global network of Pasteur Institutes. While Pastorians focused on “the technical manipulation of disease-generating microbes and disrupting their effects on an abstract, generalized human body” (Velmet 2020, p. 220), the catholic collecting policies of the NCTC sought instead to “collect the world,” defining boundaries of interest and relevance insofar as those organisms intersected with British imperial priorities. In character, the NCTC arguably bore much closer resemblance to long-established living herbaria collections, such as those at the Royal Botanic Gardens in Kew, which functioned as a “scientific empire,” facilitating a form of “imperial economic botany” (Drayton 1990, p. 219). Indeed, it is worth noting that the establishment of the NCTC came in the same year as the Imperial Bureau of Mycology was established at Kew, with explicit references to its engagement with existing collections and biological imperialism in British India.^95^

In the decades following its founding the NCTC both shaped microbiological practice and served as a model for similar national collections internationally (Russell 2016). Understanding its origins, development, and influence reveals previously hidden features underpinning the structure and organization of 20th-century microbiology. But we can also see reflections of far broader features of scientific practice. Through a more comprehensive understanding of culture collections we learn more about the privileged position of British networks of scientific influence, the role of such networks in setting scientific research agendas, and the significance of debates about how pathogens were both classified and presented to the scientific community. As Claas Kirchhelle and Charlotte Kirchhelle have argued for a later period in the context of phage-typing: “culture collections […] underpinned important advances in scientists’ understanding of microbial diversity and infection control efforts. However, embedded geopolitics, extractive microbial sampling, and cultural biases also distorted typing efforts and resulting findings in favour of high-income countries” (Kirchhelle and Kirchhelle 2024, p. 292).

Similar features can be readily seen in the nascent national-level collections of microbes. However, as Bowker and Star have argued, since “the only good classification is a living classification,” it might be reasonable for us to regard the uncertainty and bet-hedging of the early NCTC catalog-makers—just as we might the Society of American Bacteriologists and their early attempts at classificatory frameworks—as attempting to keep doors open to change in their organization of the microbial world (Bowker and Star 2000, p. 326).

The early activities of the NCTC were founded on extant transnational connections within communities of microbiologists. In this way, we see both the NCTC and its host—the Lister Institute—as being deeply embedded within, and reliant upon, imperial structures of expertise. The aggregated collections were representative of a very distinctive and specific set of microorganisms: those which were deemed useful and important in the context of the British Empire, both in terms of disease and industry. The NCTC therefore benefitted from, and reinforced, a modified international network of researchers working with microorganisms. Samples of bacteria, protozoa, and fungi traversed the globe and were concentrated within a single catalog, showcasing the breadth of microscopic organisms and reinforcing their ubiquity. At the same time, the form of the initial catalog was subject to considerable debate, foreshadowing the protracted nature of discussions and controversy about microbial classification. Discussions between key figures in the shaping of British biological and medical science in this critical phase of reconstruction of scientific activities in the aftermath of World War One is equally revealing. They make visible a plurality of strong views which belie the official self-representation of the NCTC catalog: an authoritative and single source of truth about the nature and properties of, and relations between, a huge swathe of microorganisms. At the same time the microbes represented within the catalog were necessarily restricted to those originating from within networks of medics and microbiologists limited by imperial science.

This article has established some key features of the NCTC’s foundation and early operation, most especially its alignment with existing institutions and networks of British imperial science. This clears the way for further, more expansive studies, which situate it in the context of an international community of diverse culture collections and explore how this and other collections influenced and were influenced by key trends in biological and health sciences.

The account of the NCTC as a repository of standardized organisms does not sit in isolation. Like the *Drosophila* stock centers investigated by Jenny Bangham, and living botanical collections, culture collections are working collections; that is, they perpetually reproduce their contents. However, there is a crucial difference: collections of microbes, aided by vacuum drying processes refined during the early 1930s and changed only minimally since, can also exist in a state of suspended animation, needing only to reculture strains as they become unviable (Bangham 2019). For similar reasons they are again quite different from natural history type specimen collections, serving as replicable, living entities, though with comparable risks of degradation (Daston 2004). Practical limitations about precisely which microorganisms could be cultivated under laboratory conditions—some being far more fastidious than others—also renders these collections far more reliant on the extent of technical capabilities and resources than many others. For example, one of two causative organisms of leprosy, *Mycobacterium leprae*, is an obligate intracellular parasite, and cannot be straightforwardly cultured without a host, much less maintained in a freeze-dried state like other strains held in culture collections (Lahiri and Adams 2016).

An additional distinctive feature of culture collections such as the NCTC is the inclusion of strains which long predate the advent of antibiotics, and which offer a window into a historic biological landscape. Nevertheless, identifying the conditions under which bacteria remained viable was a source of difficulty, experienced not only by the NCTC but other culture collections, such as the National Collection of Plant Pathogenic Bacteria which was established formally in the late 1940s. Just as Jacalyn Duffin has, in her study of transnational networks in postwar culture collections, begun to focus on the case of Canadian microbiologist Stanley Morris Martin in the 1960s and 1970s, we can see the intersection between culture collections and cultures of collecting as a process of refining the long-established natural historical traditions of determining the nature, significance and organization of type specimens (Duffin 2024).

At the same time, these collections reinforce the complex processes operating around the reproducibility of modern scientific practice. What we see from the very early stages of the NCTC is that the very things being reproduced were themselves a reflection of a particular construction of science, laced with politics and the networks of imperial power. Far from the “open sharing of knowledge and organisms” which Strasser invokes for the operation of the American Type Culture Collection (Strasser 2019, p. 65), we should instead see these collections as extensions of central management and control over the microbial world, in the case of the NCTC being infused with the priorities of a select coterie of individuals and institutions. The fact that donors of strains to the collection were treated favorably, with costs for the issuing of other strains to them waived as a form of thanks, indicates that institutions with greater capacity to identify and deposit novel organisms were in a privileged position compared to smaller, less well-equipped laboratories. That culture collections functioned for a long period as arbiters in microbial systematics, defining the arrangements and limits of microbe species and their relationship with the plant and animal kingdoms, reinforces the need for us to better understand how and why they operated in the ways that they did. Further consideration of the impact of this and other culture collections, whether of microorganisms such as bacteria, fungi and viruses, or stem cells, therefore has the potential to reveal far more precisely the extent to which key aspects of 20th century bioscience were a direct product of the priorities of such collections, the individuals and institutions which sat behind them, and the networks of which they were a key constituent part.

## Data Availability

No datasets were generated or analysed during the current study.

## Footnotes

1 World Data Centre for Microorganisms, *Culture Collections Information Worldwide*, https://ccinfo.wdcm.org/ (accessed March 14, 2025).

2 Crucially, however, the American Type Culture Collection was established with only around 175 strains (Strasser 2019, p. 65), remarkably similar in scale to the Lister Institute’s informal collection prior to 1920.

3 “Reconstruction Committee,” HC Deb July 10, 1916, vol 84 cc33-4.

4 The Medical Research Committee was, in 1920, reorganized and renamed the Medical Research Council, with a more wide-ranging role in directing medical research within the United Kingdom (Landsborough Thomson 1987).

5 “Minute Book of the MRC,” October 19, 1916, The National Archives (hereafter: TNA), FD 6/2. The term “public interest” perhaps reflects the wider focus on scientific research of practical utility, demonstrated by the MRC’s extensive support for projects on military health alongside more theoretical investigations during its early years of operation (Landsborough Thomson 1987).

6 “Minute Book of the MRC,” November 9, 1916, and “Minute Book of the MRC,” December 16, 1916, TNA, FD 6/2.

7 Walter Fletcher, “Proposals for a National Collection of Cultures of Bacteria,” November 20, 1919, TNA, FD 1/985.

8 A. E. Boycott to C. J. Martin, July 26, 1919, TNA, FD 1/985. As the NCTC’s first Curator, Ralph St. John-Brooks, noted in his historical reflections, further broad collections existed elsewhere at the American Museum of Natural History in New York, with “what might be called specialist collections,” such as the *Centraal-bureau voor Schimmelcultures* at Baarn, Holland, and the associated *Laboratorium voor Microbiologies* at Delft, the Collection of the *Carlsberg Laboratorium* at Valby, near Copenhagen, which concerned itself with the maintenance of yeasts, and the Collection of the *Institut für Gärungsgewebe* in Berlin. The holdings at the American Museum of Natural History would later become the basis for the American Type Culture Collection in 1925 (R. St. John-Brooks 1944, p. 284).

9 Charles-Edward Amory Winslow, “Bacteriological Collection and Bureau for the Distribution of Bacterial Cultures,” n.d. [circa 1913], New York: Museum of Natural History, Box 4. In National Collection of Type Cultures archives, UK Health Security Agency, Colindale, London (hereafter: NCTC).

10 C. J. Martin to Walter Fletcher, September 26, 1919, TNA, FD 1/985.

11 Walter Fletcher to C. J. Martin, November 20, 1919, TNA, FD 1/985.

12 Walter Fletcher to C. J. Martin, November 20, 1919, TNA, FD 1/985.

13 C. J. Martin to Walter Fletcher, November 22, 1919, TNA, FD 1/985.

14 Walter Fletcher to C. J. Martin, November 20, 1919, TNA, FD 1/985. Fletcher affirmed in later correspondence that the Lister Institute had provided such supply services over many years previously. As he later wrote to Martin: “The Lister Institute, as the [Medical Research] Committee are well aware, have long given much assistance of this kind to workers by sending standard cultures from their collection upon demand” (Walter Fletcher to C. J. Martin, December 3, 1919, TNA, FD 1/985).

15 Walter Fletcher to C. J. Martin, November 20, 1919, TNA, FD 1/985.

16 Walter Fletcher to C. J. Martin, November 20, 1919, TNA, FD 1/985.

17 Walter Fletcher to C. J. Martin, November 20, 1919, TNA, FD 1/985.

18 Walter Fletcher to C. J. Martin, November 20, 1919, TNA, FD 1/985.

19 Martin framed the enterprise as “a representative collection for the use of pathologists” (C. J. Martin to Walter Fletcher, November 22, 1919, TNA, FD 1/985).

20 C. J. Martin to Walter Fletcher, November 27, 1919, TNA, FD 1/985.

21 “Minute Book of the MRC,” November 28, 1919, TNA, FD 6/2.

22 The precise scope of “protist” was itself fluid and contested, varying in scale from Ernst Haeckel’s proposed third kingdom alongside animals and plants in 1866 to subsequent revisions and refinements in which kinds of unicellular organisms might be best considered part of the group (Haeckel 1866; Scamardella 1999). The designation of infusoria – only later protista – as independent single-celled organisms was largely settled by the mid-1870s (Churchill 1989, p. 213). However, the nature of these organisms, as well as microbes and fungi, in relation to other kingdoms in general was hotly debated, with systematists arguing back and forth about whether they were best suited to either the principles of plant or animal taxonomies.

23 Walter Fletcher to C. J. Martin, December 3, 1919, TNA, FD 1/985.

24 Walter Fletcher to C. J. Martin, December 3, 1919, TNA, FD 1/985.

25 Interestingly, in keeping with the sparse references to the National Collection of Type Cultures dotted around the historical literature, the entry for Ledingham in the *ODNB* notes that he “took part in establishing the national collection of type cultures” in 1920 (Clegg and O’Neill 2004). This amounts to a rather ambiguous description of Ledingham’s role, which was directly conferred on him by the MRC and Lister Institute, though he was undoubtedly instrumental in operationalizing the NCTC in its early phase.

26 Walter Fletcher to C. J. Martin, December 3, 1919, TNA, FD 1/985.

27 C. J. Martin to Walter Fletcher, December 16, 1919, TNA, FD 1/985.

28 Charles Ledingham to Walter Fletcher, January 7, 1920, TNA, FD 1/985. Ledingham had evidently interviewed St. John-Brooks in December 1919, since St. John-Brooks wrote to request clarification about whether his employment would come directly through the MRC, and therefore be accompanied by “the same status with regard to pension and so forth as other Medical Officers on the Staff of the Committee.” He also included his curriculum vitae (R. St. John-Brooks to Charles Ledingham, December 16, 1919, TNA, FD 1/985).

29 Charles Ledingham to Walter Fletcher, January 7, 1920, TNA, FD 1/985.

30 Charles Ledingham to Walter Fletcher, January 7, 1920, TNA, FD 1/985.

31 “National Collection of Type Cultures (Medical Research Committee) Provisional Constitution and Regulations for Approval,” [January 1920], TNA, FD 1/985.

32 “National Collection of Type Cultures (Medical Research Committee) Provisional Constitution and Regulations for Approval,” [January 1920], TNA, FD 1/985.

33 “National Collection of Type Cultures (Medical Research Committee) Provisional Constitution and Regulations for Approval,” [January 1920], TNA, FD 1/985.

34 Charles Ledingham to Walter Fletcher, January 21, 1920, TNA, FD 1/985.

35 “Minute Book of the MRC,” January 23, 1920, TNA, FD 6/2.

36 “National Collection of Type Cultures: Suggested Constitution and Regulations,” January 23, 1920, TNA, FD 1/985.

37 Equally revealingly, the letter was headed “Central Collection of Type Cultures of Bacteria and Protozoa.” Walter Fletcher to Robert Morant, February 4, 1920, TNA, FD 1/985.

38 Walter Fletcher to Robert Morant, February 4, 1920, TNA, FD 1/985.

39 Walter Fletcher to Charles Ledingham, February 10, 1920, TNA, FD 1/985.

40 Walter Fletcher to Ralph St. John-Brooks, February 11, 1920, TNA, FD 1/985; Walter Fletcher to Mabel Rhodes, February 11, 1920, TNA, FD 1/985. The circumstances surrounding Mabel Rhodes’s appointment are made apparent in communications between Martin and Fletcher around this time. She was already employed by the Lister Institute, in part as an illustrator “making drawings of microscopical preparations etc. to illustrate papers by members of staff” (C. J. Martin to Walter Fletcher, February 13, 1920, TNA, FD 1/985). With Fletcher’s permission, conferred via the MRC Assistant Secretary, Arthur Landsborough Thomson, she continued in this work on an occasional basis alongside her employment at the NCTC (A. Landsborough Thomson to C. J. Martin, February 17, 1920, TNA, FD 1/985).

41 One of the strains from Harden’s collection – then classified as *Zygosaccharomyces barkeri*, now *Saccharomyces cerevisiae* – remains, albeit now within the specialist National Collection of Yeast Cultures (National Collection of Yeast Cultures 2025).

42 The most comprehensive obituary of St. John-Brooks contains some inaccuracies, which can be corrected by consulting the outline of his career which he submitted in connection to his appointment at the NCTC (Allen 1966).

43 R. St. John-Brooks to Walter Fletcher, February 12, 1920, TNA, FD 1/985.

44 There was a strong culture of women-led science at the Lister Institute, which included close collaborations between Chick and the likes of Elsie Dalyell, Margaret Hume, and Hannah Henderson Smith in 1919 at precisely the time when Rhodes was assuming her role within the NCTC (Chick, Hume and MacFarlane 1971). This was in addition to bacteriologist Muriel Robertson (1883–1973), who contributed a large collection on anaerobic bacteria as part of the NCTC’s original strains.

45 Charles Ledingham to Walter Fletcher, March 8, 1922, TNA, FD 1/986.

46 [Cowan], untitled typescript, c.1947, Box 2, NCTC, pp. 1–2. Although this is an anonymous and untitled typescript, its production coincides with Cowan’s arrival, and it is straightforwardly attributable to him.

47 Though this was Rhodes’s responsibility, it is clear from the varieties of handwriting that St. John-Brooks was also involved in recording the issue of sub-cultures.

48 While the records of some of these early NCTC strains, which were accessioned in numerical order, are lost, we can be certain that Kidd received samples of these particular microbes. The information for these comes from historical deposit cards held at the NCTC, indicating the provenance and, in some cases, properties, of the strain (“Reference No. 11;” “Reference No. 12;” “Reference No. 13;” “Reference No. 14;” deposit cards, n.d., “Despatch Book No.1” (1920–1923), uncataloged, NCTC.

49 “Reference No. 11;” “Reference No. 12;” “Reference No. 13;” “Reference No. 14;” uncataloged deposit cards, n.d., NCTC, UKHSA. For example, from data within the present-day NCTC catalog, we can see that strain NCTC 3, *Shigella flexneri*, so-called “Oxford Flexner,” was accessioned on January 1, 1920, and was therefore already held by the Lister Institute (UK Health Security Agency. n.d. Bacteria Collection: NCTC 3 Shigella flexneri. https://www.culturecollections.org.uk/products/bacteria/detail.jsp?refId=NCTC+3&collection=nctc. Accessed March 14, 2025). The strain was deposited by the noted British bacteriologist F. W. Andrewes.

50 “Despatch Book No.1” (1920–1923), uncataloged, NCTC.

51 “Reference No. – 76B”, uncataloged card record, NCTC.

52 “Despatch Book No.1” (1920–1923), uncataloged, NCTC. Interestingly, while samples of *Vibrio cholerae* – the causative agent of cholera – were well represented in the early stages of the NCTC’s existence (they constituted specimens numbered 17 through to 29), very few requests for these highly virulent organisms were forthcoming. Most of these strains had, in fact, been preserved in the Lister Institute’s own collection, with only two of the thirteen strains having a different, and unknown, provenance.

53 “Despatch Book No.1” (1920–1923), uncataloged, NCTC.

54 “Despatch Book No.1” (1920–1923), uncataloged, NCTC.

55 A. Landsborough Thomson to Henry Dale, April  27, 1920, TNA, FD 1/985; A. Landsborough Thomson to Bulloch, April  27, 1920, TNA, FD 1/985; W. H. Bulloch to Walter Fletcher, April 28, 1920, TNA, FD 1/985.

56 The announcements also confirmed that, if sufficient strains of individual species were to be received, the NCTC would focus on “studies in classification,” as well as “[s]ubsidiary researches on the viability of bacteria in artificial media” (Anon. 1920b, p. 682).

57 R. St. John-Brooks to A. Landsborough Thomson, May [16], 1920, TNA, FD 1/985.

58 A. Landsborough Thomson, “[Typewritten Note],” May 31,  1920, TNA, FD 1/985. An exception to this was the short announcement which appeared in July 1920 in *Nature* (Anon. 1920c, p. 594).

59 A. Landsborough Thomson to Charles Ledingham, July 5, 1920, TNA, FD 1/985.

60 Charles Ledingham, “National Collection of Type Cultures,” August [4], 1920, TNA, FD 1/985. The short report also noted that the reach of the collection had begun to expand beyond those initial networks, with a reciprocal agreement of exchange established with the American Museum of Natural History, and samples received from continental Europe in addition to those arriving from “Great Britain and the Empire.”

61 The institutions included Yale University, the Bombay Bacteriology Laboratory, and various municipal research laboratories. “Despatch Book No.1” (1920–1923), uncataloged, NCTC.

62 Charles Ledingham to Walter Fletcher, February 2, 1921, TNA, FD 1/985.

63 [Ralph St. John-Brooks], “The Work of the National Collection of Type Culture,” [1939], Box 2, NCTC.

64 “Despatch Book No.1” (1920–1923), uncataloged, NCTC.

65 Walter Fletcher to Charles Ledingham, October 12, 1922, TNA 1/986; Charles Ledingham to Walter Fletcher, October 9, 1922, TNA, FD 1/986.

66 Mabel Rhodes, “The National Collection of Type Cultures,” c.1947, p. 2, Box 4, NCTC.

67 S. P. Lapage “The National Collection of Type Cultures,” c.1971, p. 4, FD 7/230, TNA.

68 This was noted in the first book recording strain distributions: “Despatch Book No.1” (1920–1923), uncataloged, NCTC, p. 1. This occurred over two years before the first catalog was published and so it is likely that Wilson’s existing professional connections, most likely St. John-Brooks or an intermediary at the RAMC, made him aware of which strains might be available.

69 Graham S. Wilson, “Notebook recording work on bacterial isolates: cultural characteristics, 1919, 1920, 1924, 1925, 1926,” 1919–1926, pp. 55 and 65, Wellcome Collection, PP/GSW/B/1.

70 Graham S. Wilson, “Notebook recording work 1924–1939, ‘General’,” 1924–1939, Wellcome Collection, PP/GSW/B/2.

71 “Despatch Book No.1” (1920–1923), uncataloged, NCTC.

72 Graham S. Wilson, “Notebook recording work on bacterial isolates: cultural characteristics, 1919, 1920, 1924, 1925, 1926,” 1919–1926, Wellcome Collection, PP/GSW/B/1.

73 R. St. John-Brooks to A. Landsborough Thomson, June 7, 1921, TNA, FD 1/985.

74 A. Landsborough Thomson to R. St. John-Brooks, June 22, 1921, TNA, FD 1/985. Sadly the materials surviving do not include these first draft arrangements, so it is unclear to which branches of science Landsborough Thomson was referring.

75 A. Landsborough Thomson to R. St. John-Brooks, June 22, 1921, TNA, FD 1/985.

76 A. Landsborough Thomson to W. H. Bulloch, June 23, 1921, TNA, FD 1/985.

77 Edgar Schuster to Walter Fletcher, June 30, 1921, TNA, FD 1/985.

78 Edgar Schuster to Walter Fletcher, June 30, 1921, TNA, FD 1/985.

79 Walter Fletcher to Charles Ledingham, July 4, 1921, TNA, FD 1/985.

80 R. St. John-Brooks to Charles Ledingham, July 6, 1921, TNA, FD 1/985.

81 R. St. John-Brooks to Charles Ledingham, July 6, 1921, TNA, FD 1/985.

82 Charles Ledingham to Walter Fletcher, July 6, 1921, TNA, FD 1/985.

83 Walter Fletcher to Charles Ledingham, July 22, 1921, TNA, FD 1/985.

84 Walter Fletcher to W. H. Bulloch, July 22, 1921, TNA, FD 1/985.

85 W. H. Bulloch to Walter Fletcher, July 25, 1921, TNA, FD 1/985.

86 Edgar Schuster to Walter Fletcher, July 26, 1921, TNA, FD 1/985.

87 Clifford Dobell to Walter Fletcher, August 4, 1921, TNA, FD 1/985.

88 Clifford Dobell to Walter Fletcher, August 4, 1921, TNA, FD 1/985.

89 Walter Fletcher to Charles Ledingham, August 1,1921, TNA, FD 1/985.

90 Walter Fletcher to Edgar Schuster, September 20, 1921, TNA, 1/985; Charles Ledingham to Walter Fletcher, August 30, 1921, TNA, 1/985; R. St. John-Brooks to Walter Fletcher, September 1, 1921, TNA, 1/985.

91 The naming of these strains indicates an occupational hazard of consulting the catalogs: their constantly shifting nomenclature. For clarity: *Bacillus pestis*, the causative organism of Bubonic Plague, was subsequently renamed *Pasteurella pestis* in the second catalog edition in 1925 (though both names persisted even in the third edition in 1931, and it is now known as *Yersinia pestis)*; *Bacillus paratyphosus C* was framed in the second edition as “*Salmonella* type” before being equated in the third edition with *Salmonella Kunzendorf* (it is now classified as one of three serovars of *Salmonella enterica*); and *Bacillus dysenteriae* of the Flexner type was subsequently given the alternative designation of *Eberthella paradysenteriae* in 1925 before being equated with *Shigella paradysenteriae* in the 1931 edition. These three examples of co-existing, shifting identities reflect the lack of consensus in microbial taxonomy and nomenclature, further revealing the importance of decision-making amongst the NCTC and MRC staff responsible for producing the catalogs (Medical Research Council 1925; Medical Research Council 1931).

92 Sadly, there are no extant records indicating which strains might have been refused inclusion. An accompanying note within the first catalog, however, noted that it was ultimately “the discretion of the Director to decide whether given cultures are of sufficient important,” a responsibility which in practice Ledingham as Director devolved to St. John Brooks as Curator (Medical Research Committee 1922, p. 6).

93 This was followed, two years later in 1925, but the first volume on bacterial systematics by R. E. Buchanan who, with St. John-Brooks as a willing collaborator, would later exert a huge influence on bacterial taxonomy through the publication of the Bacteriological Code in 1948 (Buchanan 1925).

94 The majority of Buchanan’s book had been previously published as a series of ten articles in the J*ournal of Bacteriology* between 1916 and 1918.

95 Anon., “Imperial Bureau of Mycology - Inception and Contribution of India,” 1918–1927, IOR/L/E/7/955 File 1991, British Library.

## References

[CR1] Ainsworth, Geoffrey C. 1996. *Brief biographies of British mycologists*. Stourbridge: British Mycological Society.

[CR2] Allen, L. A. 1966. Obituary notice: Ralph St John-Brooks, 1884–1963. *Journal of General Microbiology* 42:165–167. 5330335 10.1099/00221287-42-2-165

[CR3] Anderson, Ephraim S., and Robert Williams. 1988. Graham Selby Wilson, 10 September 1895–5 April 1987. *Biographical Memoirs of Fellows of the Royal Society* 34:887–919. 11616119

[CR9] Anon. 1920a. A national collection of type cultures. *The Lancet*. May 15:1081–1082.

[CR10] Anon. 1920b. National collection of type cultures. *British Medical Journal*. May 15:682

[CR11] Anon. 1920c. Notes. *Nature* 105:590–594.

[CR8] Anon. 1922. The national collection of type cultures. *The Lancet*. March 11:492–493.

[CR7] Anon. 1923. Mystery of new disease. Three victims at Lister Institute. First British cases. *Daily Chronicle *[London], January 19, p. 5.

[CR6] Anon. 1925. The progress of science. A menagerie of microbes. National collection. *The Times*, November 16, p. 8.

[CR5] Anon. 1933. Sir Walter Morley Fletcher. 1873–1933. *Obituary Notices of Fellows of the Royal Society* 1:153–163.

[CR4] Anon. 1934. Our ceaseless war against disease. *Hull Daily Mail*, December 14, p. 9.

[CR12] Bangham, Jenny. 2019. Living collections: Care and curation at *Drosophila* stock centres. *BJHS Themes* 4:123–147. 32133157 10.1017/bjt.2019.14 PMC7056353

[CR13] Bergey, David H. 1923. *Bergey’s manual of determinative bacteriology: A key for the identification of organisms of the class schizomycetes*. Baltimore: The Williams and Wilkins Company.

[CR14] Bowker, Geoffrey C., and Susan Leigh Star. 2000. *Sorting things out: Classification and its consequences*. Cambridge, Mass: MIT Press.

[CR15] Buchanan, Robert Earle. 1925. *General systematic bacteriology: History, nomenclature, groups of bacteria*. Baltimore: Williams and Wilkins Company.

[CR16] Clegg, Hugh. 2004. Ledingham, Sir John Charles Grant (1875–1944). Rev. Tim O’Neill. In *Oxford dictionary of national biography*. 10.1093/ref:odnb/34462.

[CR17] Chakrabarti, Pratik. 2012. *Bacteriology in British India*. Rochester: University of Rochester Press.

[CR18] Chick, Harriette, Margaret Hume, and Marjorie MacFarlane. 1971. *War on disease: A history of the Lister Institute*. London: A. Deutsch.

[CR19] Churchill, Frederick B. 1989. The guts of the matter. Infusoria from Ehrenberg to Bütschli: 1838–1876. *Journal of the History of Biology* 22:189–213. 11608945 10.1007/BF00139512

[CR20] Cooter, Roger. 2004. The rise and decline of the medical member: Doctors and Parliament in Edwardian and interwar Britain. *Bulletin of the History of Medicine* 78:59–107. 15161087 10.1353/bhm.2004.0012

[CR21] Daston, Lorraine. 2004. Type specimens and scientific memory. *Critical Inquiry* 31:153–182.

[CR23] Drayton, Richard. 1990. *Nature’s government: Science, imperial Britain, and the ‘improvement’ of the world*. New Haven: Yale University Press.

[CR22] Duffin, Jacalyn. 2024. Microbial culture collections: Stanley Morris Martin, the first international conference (Ottawa 1962), and beyond. *Journal of Medical Biography*. 10.1177/09677720241266311. 10.1177/09677720241266311 39155577

[CR24] Fazal, Mohammed-Abbas, Juandem Agendia, Kazutomo Yokoya, et al. 2019. National collection of type cultures: The bacteriophage and plasmid collections and repositories. *Access Microbiology*. 10.1099/acmi.ac2019.po0308.

[CR25] Felix, Arthur M., and Mabel Rhodes. 1931. Serological varieties of typhus fever. *Journal of Hygiene* 31:225–246. 20475090 10.1017/s0022172400010780 PMC2170603

[CR26] Fry, Geoffrey K. 2009. Morant, Sir Robert Laurie (1863–1920). In *Oxford dictionary of national biography*. 10.1093/ref:odnb/35096.

[CR27] Gradmann, Christoph. 2009. *Laboratory disease: Robert Koch’s medical bacteriology*. Translated by Elborg Forster. Baltimore: Johns Hopkins University Press.

[CR28] Grote, Mathias. 2017. Petri dish versus Winogradsky column: A *longue durée* perspective on purity and diversity in microbiology, 1880s–1980s. *History & Philosophy of the Life Sciences*. 10.1007/s40656-017-0175-9. 10.1007/s40656-017-0175-9 29188459

[CR29] Haeckel, Ernst. 1866. *Generelle morphologie der organismen. Allgemeine grundzüge der organischen Formen-wissenschaft, mechanisch begründet durch die von Charles Darwin reformirte Descendenztheorie*. Berlin: G. Reimer.

[CR30] Hardy, Anne. 2015. *Salmonella infections: Networks of knowledge, and public health in Britain, 1880–1975*. Oxford: Oxford University Press.

[CR31] Hirschfeld, L. 1919. A new germ of Paratyphoid. *The Lancet* 193:296–297.

[CR32] Holmes, Barry. 2018. The history of the national collection of type cultures (NCTC) and the collection as a resource for systematists and the wider scientific community. *The Bulletin of BISMiS* 7:22–43.

[CR33] Homei, Aya, and Michael Worboys. 2013. *Fungal disease in Britain and the United States, 1850–2000: Mycoses and modernity*. Basingstoke: Palgrave Macmillan. 24228294

[CR35] Kirchhelle, Claas, and Charlotte Kirchhelle. 2024. Northern normal: Laboratory networks, microbial culture collections, and taxonomies of Power (1939–2000). *Engaging Science, Technology and Power* 10:292–336.

[CR36] Kollmer, Charles A. 2022. International culture collections and the value of microbial life: Johanna Westerdijk’s fungi and Ernst Georg Pringsheim’s algae. *Journal of the History of Biology* 55:59–87. 35258710 10.1007/s10739-022-09669-6

[CR37] Kollmer, Charles A. 2020. From elephant to bacterium: Microbial culture techniques and chemical orders of nature, 1875–1946. Unpublished PhD *diss.*, Princeton University.

[CR38] Lahiri, R. and L. B. Adams. 2016. Cultivation and viability determination of *Mycobacterium leprae*. In *International textbook of leprosy* ed. D. Scollard and T. Gillis eds., ch. 5.3. Greenville SC: American Leprosy Missions. https://internationaltextbookofleprosy.org/chapter/assays-determining-viability-mycobacterium-leprae. Accessed March 14, 2025.

[CR39] Landsborough Thomson, A. 1987 (1973). *Half a century of medical research: Volume one: Origins and policy of the Medical Research Council (UK)*. London: Medical Research Council.

[CR40] Martin, C.J. 1939. Arthur Edwin Boycott. 1877–1938. *Obituary notices of Fellows of the Royal Society.* 2:560–571.

[CR41] Medical Research Council. 1922. *Catalogue of the national collection of type cultures*. London: HMSO.

[CR42] Medical Research Council. 1925. *Catalogue of the national collection of type cultures*. London: HMSO.

[CR43] Medical Research Council. 1931. *Catalogue of the national collection of type cultures*. London: HMSO.

[CR44] Medical Research Council. 1958. *The national collection of type cultures: Catalogue of species*. London: HMSO. 13503424

[CR45] Méthot, Pierre-Olivier. Bacterial transformation and the origins of epidemics in the interwar period: The epidemiological significance of Fred Griffith’s “transforming experiment.” *Journal of the History of Biology* 49:311–358. 10.1007/s10739-015-9415-6 26294287

[CR46] Morgan, Kenneth O. 2011. Addison, Christopher, first Viscount Addison (1869–1951). *Oxford Dictionary of National Biography*10.1093/ref:odnb/30342.

[CR100] National Collection of Yeast Cultures. 2025. NCYC 70 – *Saccharomyces cerevisiae*. https://www.ncyc.co.uk/catalogue/saccharomyces-cerevisiae-(73). Accessed 20 February 2025.

[CR47] Oren, Aharon. 2024. On validly published names, correct names, and changes in the nomenclature of phyla and genera of prokaryotes: A guide for the perplexed. *NPJ Biofilms and Microbiomes*. 10.1038/s41522-024-00494-9. 10.1038/s41522-024-00494-9 PMC10928132 38467688

[CR48] Parkar, M.T. 1988. Obituary notice: Graham Selby Wilson, 1895–1987. *Journal of Medical Microbiology* 25:301–304. 3282076 10.1099/00222615-25-4-301

[CR49] Public Health England. 2015. National collection of type cultures: Authenticated bacterial reference and type strains. https://www.culturecollections.org.uk/media/103033/nctc-brochure-4mb-final.pdf. Accessed 19 February 2024.

[CR50] Rhodes, Mabel. 1950. Preservation of yeasts and fungi by desiccation. *Transactions of the British Mycological Society* 33:35–39.

[CR51] Rhodes, Mabel, and P. J. Fisher. 1950. Viability of dried bacterial cultures. *Journal of General Microbiology* 4:450–456. 14778951 10.1099/00221287-4-3-450

[CR52] Russell, Julie E. 2016. Evolving a national culture collection to meet current challenges in microbiology. *Culture* 36. https://assets.thermofisher.com/TFS-Assets/MBD/Reference-Materials/Culture-36-2-Evolving-a-National-Culture-Collection-LT2299A.pdf. Accessed 14 March 2025.

[CR53] Scamardella, J. M. 1999. Not plants or animals: A brief history of the origin of Kingdoms Protozoa, Protista and Protoctista. *International Microbiology* 2:207–216. 10943416

[CR54] Smith, Homer F. 1921. Preservation of stock cultures of bacteria by freezing and drying. *Journal of Experimental Medicine* 33:69–75. 19868481 10.1084/jem.33.1.69 PMC2128170

[CR57] Sankaran, Neeraja. 2010. Mutant bacteriophages, Frank Macfarlane Burnet, and the changing nature of “genespeak” in the 1930s. *Journal of the History of Biology.* 43:571–599. 20665082 10.1007/s10739-009-9201-4

[CR58] Sexton, Christopher. 1999. *Burnet: A life*. South Melbourne: Oxford University Press.

[CR55] St. John-Brooks, Ralph. 1944. The national collection of type cultures. *British Medical Bulletin* 2:284–286.

[CR56] St. John-Brooks, Ralph, and Mabel Rhodes. 1923. The organisms of the fowl typhoid group. *Journal of Pathology and Bacteriology* 26:433–440.

[CR59] Strasser, Bruno. 2019. *Collecting experiments: Making big data biology*. Chicago: University of Chicago Press.

[CR60] Sutherland, Tonia. 2023. *Resurrecting the black body: Race and the digital afterlife*. Oakland: University of California Press.

[CR61] Tilley, Helen. 2011. *Africa as a living laboratory: Empire, development, and the problem of scientific knowledge, 1870–1950*. Chicago: University of Chicago Press.

[CR62] Tomes, Nancy. 1999. *The gospel of germs: Men, women and the microbe in American life*. Cambridge: Harvard University Press. 11623768

[CR66] Velmet, Aro. 2020. *Pasteur’s empire: Bacteriology and politics in France, its colonies, and the world*. New York: Oxford University Press.

[CR67] Wall, Rosemary. 2013. *Bacteria in Britain, 1880–1939*. London: Pickering & Chatto.

[CR69] Worboys, Michael. 2000. *Spreading germs: Disease theories and medical practice in Britain, 1865–1900*. Cambridge: Cambridge University Press.

[CR68] Worboys, Michael. 2007. Was there a bacteriological revolution in late nineteenth-century medicine? *Studies in History and Philosophy of Biological and Biomedical Sciences* 38:20–42. 17324807 10.1016/j.shpsc.2006.12.003

[CR70] Wright, Maureen. 2016. Developing mental health provision in West Sussex: Harold A. Kidd, first medical superintendent of Graylingwell Hospital, 1896–1926. *Southern History* 38. https://southernhistorysociety.org.uk/archive/southern-history-vol-38-2016/. Accessed 14 March 2025.

